# *Ganoderma lucidum*: Novel Insight into Hepatoprotective Potential with Mechanisms of Action

**DOI:** 10.3390/nu15081874

**Published:** 2023-04-13

**Authors:** Md Faruque Ahmad, Fakhruddin Ali Ahmad, Md. Zeyaullah, Abdulrahman A. Alsayegh, Syed Esam Mahmood, Abdullah M. AlShahrani, Mohammad Suhail Khan, Eman Shama, Alshaimaa Hamouda, Ehab Y. Elbendary, Kandil Abdel Hai Ali Attia

**Affiliations:** 1 Department of Clinical Nutrition, College of Applied Medical Sciences, Jazan University, Jazan 45142, Saudi Arabia; 2 Department Forensic Science, School of Engineering and Science, G.D Goenka University, Gurugram 122103, Haryana, India; faahmad97@gmail.com; 3 Department of Basic Medical Science, College of Applied Medical Sciences, Khamis Mushayt Campus, King Khalid University (KKU), Abha 62561, Saudi Arabia; 4 Department of Family and Community Medicine, College of Medicine, King Khalid University, Abha 62529, Saudi Arabia; 5 Department of Public Health, College of Applied Medical Sciences, Khamis Mushayt Campus, King Khalid University (KKU), Abha 62561, Saudi Arabia

**Keywords:** hepatoprotective, nutritional constituents, mechanisms, cancer, NAFLD, alcohol

## Abstract

*Ganoderma lucidum* (*G. lucidum*) has been widely used for its health benefits as an edible and traditional medicinal mushroom for thousands of years in Asian countries. It is currently used as a nutraceutical and functional food owing to its major bioactive compounds, polysaccharides and triterpenoids. *G. lucidum* exhibits a broad range of hepatoprotective impacts in various liver disorders, such as hepatic cancer, nonalcoholic fatty liver disease (NAFLD), alcohol-induced liver disease, hepatitis B, hepatic fibrosis, and liver injury induced by carbon tetrachloride (CCl4) and α-amanitin. *G. lucidum* protects the liver through a broad range of mechanisms that include the modulation of liver Phase I and II enzymes, the suppression of β-glucuronidase, antifibrotic and antiviral actions, the regulation of the production of nitric oxide (NO), the maintenance of hepatocellular calcium homeostasis, immunomodulatory activity, and scavenging free radicals. *G. lucidum* could signify an encouraging approach for the management of various chronic hepatopathies, and its potential mechanisms make it a distinctive agent when used alone or with other drugs and applied as a functional food, nutraceutical supplement, or adjuvant to modern medicine. This review summarizes the hepatoprotective properties of *G. lucidum* with its various mechanisms of action on different liver ailments. Biologically active substances derived from *G. lucidum* are still being studied for their potential benefits in treating different liver ailments.

## 1. Introduction

*G. lucidum* is a substantial source of nutritionally and pharmacologically important potential constituents. It has been used as an herbal cure for many years in traditional Japanese and Chinese medicine [1,2]. The possible pharmacologically active components and nutritional composition of this traditional treatment have captivated the curiosity of scientists and researchers wishing to investigate its usefulness [3]. It has been claimed that the mushroom species *G. lucidum* can prolong one’s life and improve health. As a source of distinctive bioactive metabolites, which grants it a number of attributes, it has been demonstrated to be efficient in the treatment and management of a variety of diseases [4]. *G. lucidum* has demonstrated potent anticancer [5,6], anti-inflammatory [7], hepatoprotective [8], antidiabetic [9], cardioprotective [10], immunomodulatory [11], antioxidant [12], and antiaging [13] activities. Due to its distinct potential as a medicine, there is increased demand in the food sector for it to be used as a significant source for nutrient supplements [14]. According to reports, the comprehensive immunomodulatory and antioxidant actions of this substance are primarily responsible for its wide range of medicinal and pharmacological effects [4,14].

The term “liver diseases” is used to describe a broad range of illnesses that prevent or impair the liver’s ability to operate. Recent studies have found that *G. lucidum* has a variety of hepatoprotective effects on liver illnesses, including alcoholic liver disease [15], viral hepatitis [16], NAFLD [4], fibrosis [17], hepatic cancer [8], CCl4 and amanitin-induced liver injury, etc. [18]. The prevalence of liver illnesses is currently increasing globally, necessitating the urgent development of preventive and treatment strategies. Therefore, a natural alternative with fewer side effects is needed. The biologically active constituents of *G. lucidum* are a promising approach to surmount such challenges and provide a potential natural hepatoprotective agent [17].

## 2. Materials and Methods

For the present comprehensive review, we collected evidence through diverse databases that included Science Direct, Saudi Digital Library, Scopus, Google Scholar and PubMed. The following keywords were used: *G. lucidum*, *G. lucidum* triterpenoids, *G. lucidum* polysaccharides (GPLS), β-glucans, D-glucans, ganoderic acids, ganoderic acid A, ganoderic acid B, ganoderic acid C1, *G. lucidum* triterpenoids extracts, hepatic fibrosis, hepatic cancer, hepatitis and hepatitis B. The following phrases were included: “*G. lucidum* hepatoprotective effects”, “Effect of *G. lucidum* in hepatic cancer”, “Effect of *G. lucidum* in hepatic fibroris”, “Effect of *G. lucidum* in NAFLD”, “Effect of *G. lucidum* in CCl4 induced hepatic injury”, “Effect of *G. lucidum* in hepatitis B”, “Effect of *G. lucidum* in formaldehyde induced hepatic injury”, “Effect of *G. lucidum* in α-Amanitin induced liver injury” and “Effect of *G. lucidum* in alcohol-induced hepatic injury”. Articles published in English were chosen to study the hepatoprotective effects of *G. lucidum* in our literature survey.

## 3. Nutritional and Bioactive Constituents

Polysaccharides and triterpenoids are two of the main biologically potent components of *G. lucidum*. Several monomers—for instance, galactose, glucose, xylose, mannose, fucose, rhamnose and arabinose—in the composition of *G. lucidum* polysaccharide have been described as significantly contributing to its antioxidant, immunomodulatory, antitumor, and antibacterial characteristics [19,20]. It is well known that polysaccharides, particularly D-glucans, can inhibit angiogenesis and also have immunomodulatory effects on tumors [8]. Additionally, polysaccharides defend against free radicals and lessen the injuries that mutagens inflict on cells. In general, polysaccharides of *G. lucidum,* particularly β-glucans, synergistically improve immune system functioning and potentiate immune cells’ anticancer activities and cytokine production, whereas *G. lucidum* triterpenes inhibit the cancer cells growth, proliferation and invasion [21,22,23]. Generally, mushroom polysaccharides serve as an essential component of the cell walls of fruiting bodies, mycelia and other portions. They exhibit potent bioactivities in hepatoprotection and anti-inflammatory properties [24,25]. The majority of mushroom polysaccharides have been discovered to be low in toxicity to the liver and even have no major side effects, which makes them a scientific hotspot in the field of natural remedies as well as in the functional food and nutraceutical industries, particularly for their hepatocellular protection [26,27].

Triterpenes, which include ganoderic acids (GAs), ganodermic acid, lucinedic acids, ganoderols and lucidones, are the most prevalent terpenes and have been linked to a variety of potential benefits against cancer, inflammation, hepatitis, hypoglycemia, microbes and HIV-1 [28,29,30]. Numerous studies have been conducted on *G. lucidum*’s phenolic composition, with phenolic acids emerging as the most significant class. These acids include chlorogenic, gallic, cinnamic, protocatechuic, p-coumaric, p-hydroxybenzoic and p-coumaric acids. Anti-inflammatory, antibacterial, antityrosinase and antioxidant activities have been linked to these compounds [31,32,33]. In addition to all of these bioactive substances, the nutritional profile of *G. lucidum* indicates a high potential for its application as a functional food and nutraceutical in various forms, such as tablets, pills and capsules [33]. Other complex compounds that have been reported in various research studies include alkaloids, germanium, nucleotides, glycoproteins, pro-vitamin D2, coumarins, lysozyme, flavonoids, enzymes, long-chain fatty acids, essential amino acids, sterols and different minerals, such as iron, copper, selenium, magnesium, zinc, potassium, phosphorus and calcium with vitamins [34,35].

**Figure 1:**
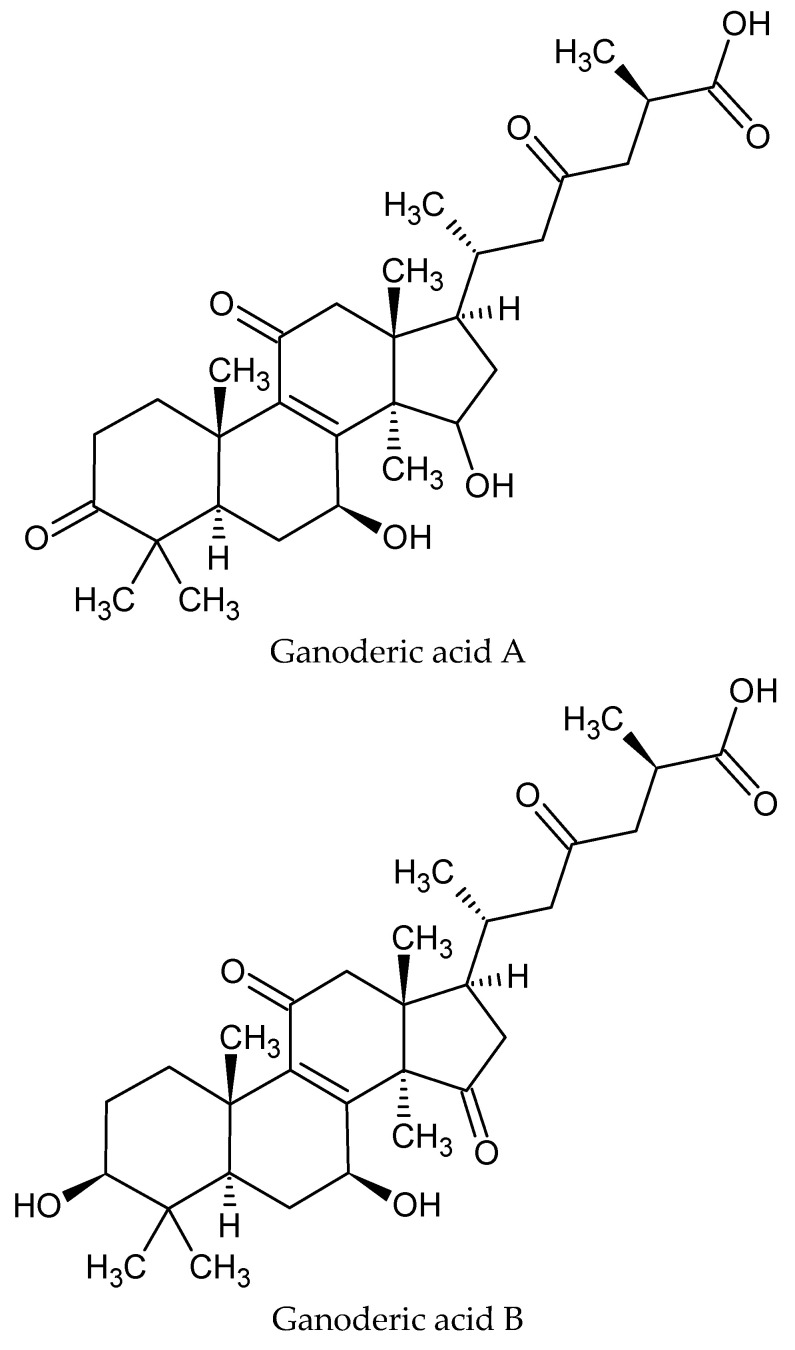

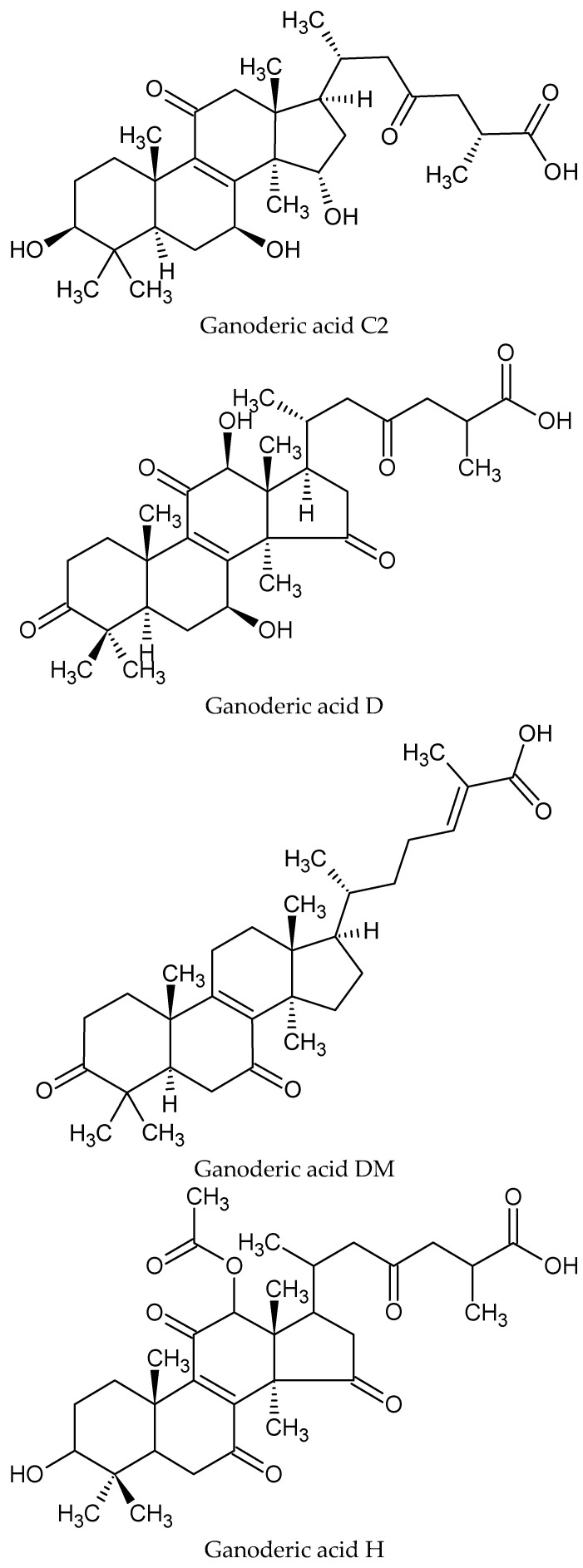

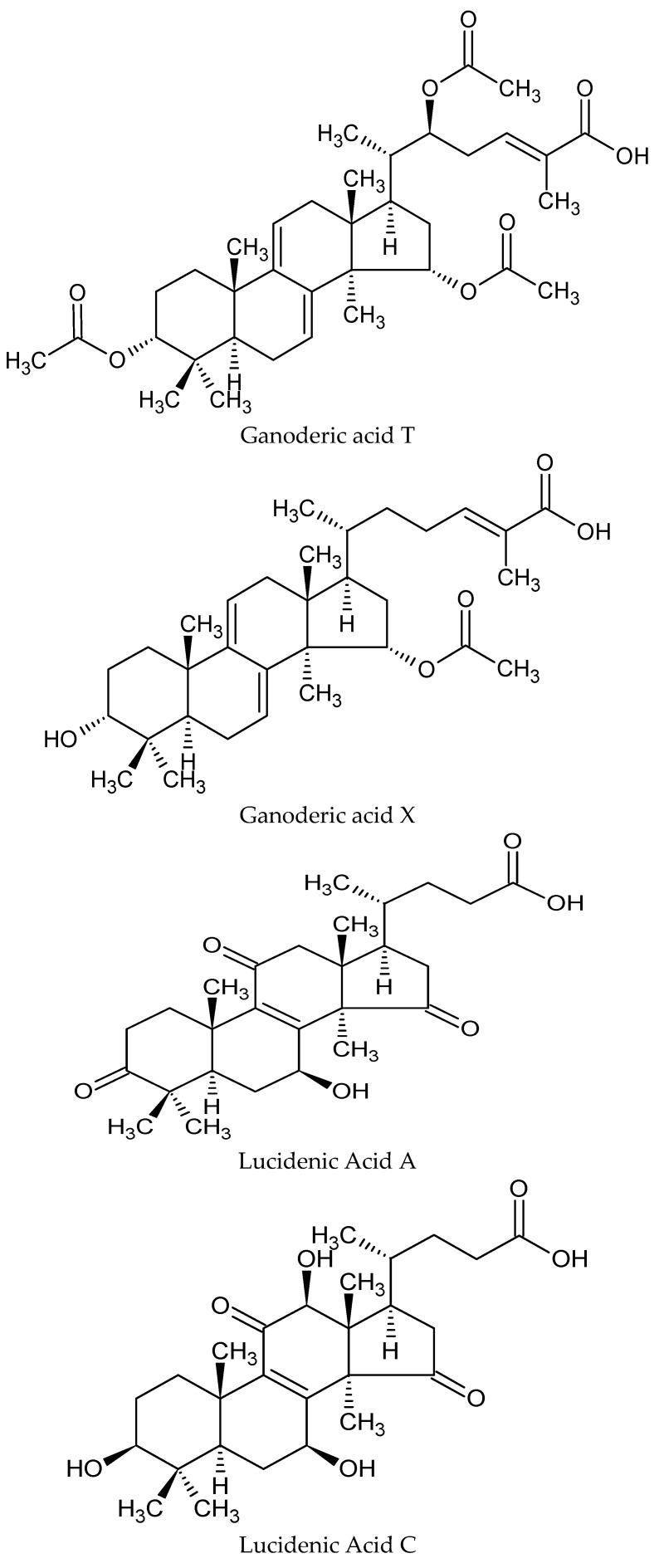

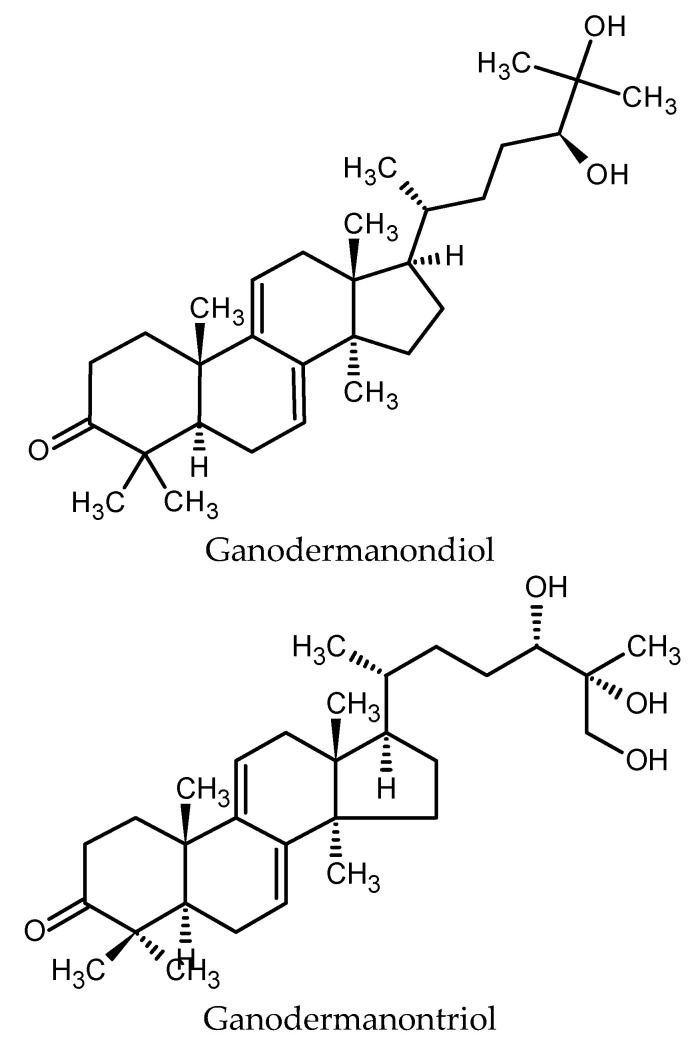
Different potential triterpenoids isolated from *G. lucidum* that reveal protective actions in various diseases.

Triterpenoids and polysaccharide-rich *G. lucidum* may influence the efficiency of its hepatoprotective effects by reducing the overabundance of free radicals and preserving cells from oxidative stress [36]. Through its antioxidant property, *G. lucidum* water extract prevented hepatic damage in mice subjected to acute hepatic injury induced by cadmium or amanitin [20,37]. Triterpenoids exhibited preventative effects against liver damage in mice that was caused by D-galactosamine, amanitin and CCl4. These triterpenoids’ hepatoprotective mode of action has also been proven [37,38]. Different formulas of *G. lucidum*, such as spore oil, sporoderm-breaking spores and spore powder have proven to be effective against hepatic ailments [15,39,40,41]. Various triterpenoids isolated from *G. lucidum* can be seen in Figure 1.

## 4. Potential Mechanisms of Action

**Figure 2:**
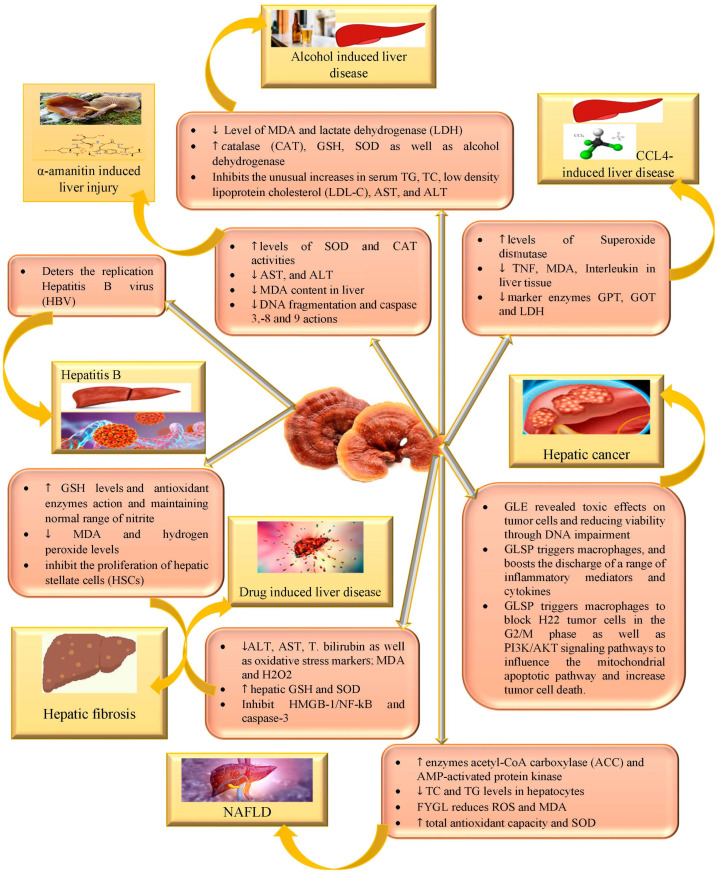
Hepatoprotective action exhibited by *G. lucidum*-isolated triterpenoids and polysaccharides against alcohol-induced hepatic injury, NAFLD, liver fibrosis, hepatic cancer, hepatitis B, α amantin, CCL4 and drug-induced hepatic injury.

*G. lucidum* is widely regarded as a potentially valuable, conventional healing approach for various types of hepatic disorders. *G. lucidum* is a medicinal mushroom that is also considered an edible mushroom. *G. lucidum* possesses immunomodulators and anticancer activities through various mechanisms of action, demonstrating profound impacts against various types of cancer and other diseases as well as hepatoprotective activity [11,42]. Immunomodulatory attributes of *G. lucidum* have been reported in a wide range of diseases due to its multifaceted mechanism of action. *G. lucidum* polysaccharide (GLPS) illustrates immunomodulatory characteristics through the enhancing action of the mononuclear phagocyte system and antigen-presenting cells, along with cellular and humoral immunity. β-glucans obtained from *G. lucidum* are predicted to exhibit an immune reaction via pathogen-associated molecular patterns (PAMPs) [43]. β-glucans through the binding Dectin-1 receptor existing on various cells, such as monocytes, macrophages, dendritic cells and neutrophils, generate signal transduction, resulting in activated T cells, mitogen-activated protein kinases and nuclear factor-κB, as well as increasing the production of cytokines and enhancing immunological response [44,45]. Additionally, there are numerous arguments in favor of compounds derived from *G. lucidum* that demonstrate anticancer properties through a variety of mechanisms, including host immune response activation, cytotoxic action on cancer cells, the suppression of angiogenesis, the downregulation of uPA and uPA receptor expression in cancerous cells and the induction of cell differentiation [46,47]. The various mechanisms of action exhibited by *G. lucidum* against different liver disorders can be seen in Figure 2.

The use of *G. lucidum* could be a good approach to protecting against a wide range of hepatic disorders. The mechanisms of *G. lucidum*’s hepatoprotective impacts are widely undefined. Nevertheless, substantial evidence suggests a number of mechanisms which include radical-scavenging behavior, antioxidant properties, the modulation of hepatic enzymes, β-glucuronidase inhibition, antiviral and antifibrotic activity, hepatocellular calcium maintenance, homeostasis, the production of nitric oxide and the impacts of immunomodulation. Its molecular mechanisms as well as bioactive constituents should be researched to further enhance the management of hepatic diseases and chemically induced hepatic complications [17,37,48,49,50]. The various activities that *G. lucidum* exhibits against different hepatic disorders are compiled in Table 1.

## 5. Potential Hepatoprotective Effects

### 5.1. Protective Effects against Liver Fibrosis

Fibrosis is a condition defined by the excessive accumulation of extracellular matrix components which can cause organ failure and death. Up to 45% of all fatalities in affluent countries are caused by fibrosis [65]. Through the TLR4/NF-kB/MyD88 signaling pathway, GLPS drastically reduces hepatic fibrogenesis and inflammation in rats. It has also been shown that GLP strongly suppresses hepatic stellate cells’ activation in mice and in TGF-1-induced HSC-T6 cells, which can be shown by decreasing the expressions of collagen I and α-SMA. RNA-sequencing has revealed that apoptosis, inflammation, the cell cycle, ECM–receptor interactions, and the TGF-β/Smad and TLR4/NF-κB signaling pathways are repressed by the administration of GLP. GLP elicits anti-fibrotic activities that have all been linked with apoptosis, the inhibition of the cell cycle, the induction of S-phase arrest in vitro and the regression of ECM–receptor interaction-related molecular expression, specifically integrins ITGA6 and ITGA8 expression [6]. 

*G. lucidum* extract (GLE) exhibits potential preventive as well as therapeutic results in studies of formaldehyde (FA)-induced liver fibrosis [63]. *G. lucidum*’s hepatoprotective effectiveness against hepatic fibrosis induced by FA was assessed by measuring aspartate aminotransferase (AST), alanine aminotransferase (ALT) and alkaline phosphatase (ALP). ALT is a crucial enzyme for liver fibrosis, which leads to catalyzing the transamination process. Liver fibrosis is directly influenced by the increase in enzyme activity [17,66]. These enzyme levels were noticeably greater in the FA group, but they were also noticeably lower after treatment with *G. lucidum*. The pathogenesis of liver fibrosis is significantly impacted by TNF, IL-1 and IL-6. TNF is primarily a group of pro-inflammatory cytokines that are known to be crucial in causing liver fibrosis. During hepatotoxic fibrosis, the liver releases IL-1, IL-6 and TNF into the blood. When FA was given to the rats, it was noted that FA significantly increased the amounts of these cytokines in their livers. The group that received 100 mg/kg of *G. lucidum* had significantly lower cytokine levels than those of the control group. These results provided evidence for the hepatoprotective properties of *G. lucidum* [63]. *G. lucidum*, through lowering malondialdehyde (MDA) and hydrogen peroxide levels, boosting glutathione (GSH) and antioxidant enzymes, and maintaining normal ranges of nitrite and myeloperoxidase formation in FA-treated rats, exhibits liver-protective effects [63].

Comprehensive research was conducted on hepatic fibrosis induced by D-galactosamine (D-GalN), and the effects of *G. lucidum* triterpenes on hepatic fibrosis were evaluated [53]. The serum marker enzyme (ALT and AST) function, levels of liver superoxide dismutase (SOD), MDA, and GSH activity were significantly increased in D-GalN-induced liver fibrosis. These parameters were maintained at their usual levels in mice pretreated with *G. lucidum* total triterpene extracts. The ideal hepatoprotective outcome for the total triterpene extract from *G. lucidum* was found at an amount of 180 mg/kg, based on biological indicators and a liver histopathology investigation [53,54]. These findings could imply that the obtained *G. lucidum* triterpenoids had a potent efficacy against D-GalN-induced hepatic fibrosis. The activity of enzymes that neutralize free radicals, thereby increasing antioxidant potential, may be associated with the hepatoprotective efficacy of *G. lucidum* triterpenoid extract. *Ganoderma* of other species was also found to be effective against hepatic fibrosis. Triterpenoids in *Ganoderma applanatumm* such as ganoapplanic acid A, ganoapplanic acids C and F, and ganoapplaniates D, inhibited the proliferation of hepatic stellate cells (HSCs) [55].

### 5.2. Protective Effects against Alcohol-Induced Liver Injury

Due to the fact that alcohol is one of the most popular psychoactive constituents, alcohol abuse and dependency are progressively becoming a crucial issue on a global scale. About 2.5 million people each year die as a result of frequent and excessive alcohol consumption [23]. One of the major dangers in the emergence of many liver illnesses is alcohol-induced liver damage. Abuse of alcohol is a pathogenic factor in 10–35% of cases of alcoholic hepatitis and 10% of cases of liver cirrhosis [67]. *G. lucidum* has a number of amazing advantages for lipid metabolism and liver health. It has been found that *G. lucidum* ethanol extract (high in GAs) exhibits a defensive effect against liver injury induced by alcohol in mice [68]. In addition to significantly protecting the liver from excessive hepatic lipid accumulation and pathological changes caused by alcohol, *G. lucidum* ethanol extract inhibits anomalous upsurges in total cholesterol (TC), serum triglyceride (TG), low-density lipoprotein cholesterol, ALT and AST. Additionally, GLE diet interventions significantly reduce MDA and lactate dehydrogenase (LDH) levels in the liver and increase catalase (CAT), GSH, alcohol dehydrogenase (ADH) and SOD levels, which all help to combat alcohol-induced oxidative stress [51]. In addition, the composition of liver metabolites in mice consuming excessive amounts of alcohol was examined using liver metabolomics profiling, and it was found that GLE interventions significantly regulated the amounts of some biochemical parameters related to primary bile acid biosynthesis, the metabolism of riboflavin and tryptophan, unsaturated fatty acid biosynthesis, and the metabolism of fructose and mannose [60]. In addition, a diet with GLE dramatically controlled the levels of mRNA for important genes linked to fatty acid metabolism, the breakdown of ethanol and the inflammatory response in the liver. These results suggest that *G. lucidum* ethanol extract has the potential to be helpful in reducing alcohol-induced liver injury [51].

In addition, a study found that GA supplementation significantly reduced abnormally elevated liver indices, serum lipid parameters, AST, ALT and lipid accumulation in mice exposed to alcohol [68]. In a specific study on ganoderic acid (GA)-A, it was reported that it showed a substantial regulatory effect on liver metabolites’ composition in alcohol-exposed mice, particularly biomarker levels linked in the metabolic pathways of riboflavin, serine, glycine, pyruvate metabolism, unsaturated fatty acid biosynthesis, the metabolism of ketone bodies, mannose and fructose. Furthermore, dietary supplementation with GA-A significantly controlled the mRNA levels of genes involved in lipid metabolism and the inflammatory response in the liver [69]. In addition, GA interventions controlled the liver’s mRNA levels of genes involved in metabolism, oxidative stress, bile acid production, and the metabolism of fatty acids, alcohol and other substances. These findings show that GA interventions can considerably reduce the effects of alcoholism on the liver, emerging as a promising novel functional nutrient for alcoholism prevention [69].

### 5.3. Protective Effects against Non-Alcoholic Fatty Liver Disease

NAFLD is the term used to describe hepatic steatosis that is not linked to increased alcohol intake or other obvious hepatotoxic factors [70]. The metabolic syndromes of obesity, diabetes, insulin resistance (IR), hypertension, atherosclerosis, dyslipidemia, systemic inflammation and others are most frequently linked with NAFLD, which is a clinicopathologically defined entity. According to studies by Pappachan JM et al. (2014) and Fazel Y et al. (2016), NAFLD affects 30% of the general population in developed nations and can reach 70% in type 2 diabetic patients or 90% in people who are severely obese. NAFLD refers to a group of pathologic alterations that start with steatosis and develop into steatohepatitis (NASH), cirrhosis and hepatocellular cancer [71,72].

Effective drugs against NAFLD are needed. Fudan-Yueyang *G. lucidum* (FYGL), a hyperbranched proteoglycan (a composition of lipophilic protein and hydrophilic polysaccharide) isolated from *G. lucidum*, inhibits the steatosis caused by palmitic acid (PA) in HepG2 hepatocytes [60]. FYGL significantly reduces TC and TG levels in hepatocytes by increasing the activity of the enzymes’ acetyl-CoA carboxylase (ACC) and AMP-activated protein kinase (AMPK), which in turn suppresses the expression of the enzymes’ sterol regulatory element-binding protein 1 (SREBP1) and fatty acid synthase. Furthermore, this prevents steatosis induced by the oxidation of fatty acids by increasing the expression of carnitine palmitoyl transferase-1 (CPT-1). In the meantime, FYGL can reduce reactive oxygen species (ROS) and MDA as well as boost overall antioxidant capacity and SOD [59]. These findings show that FYGL may have the ability to protect hepatocytes from lipid buildup, oxidative stress and apoptosis, acting as a possible NAFLD treatment. 

**Figure 3:**
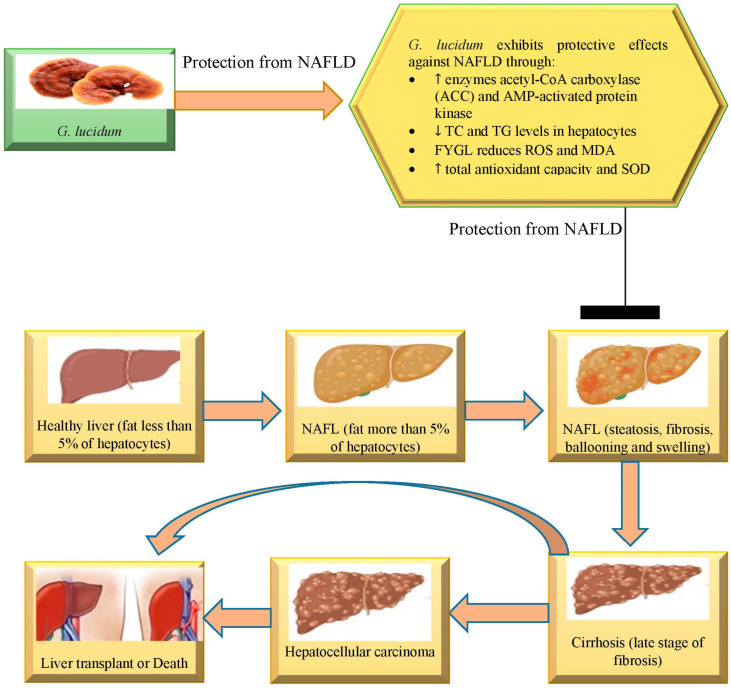
*G. lucidum* exhibits protective effects against the development of the spectrum of non-alcoholic fatty liver disease through its multifaceted mechanism.

However, other *Ganoderma* species, such *Ganoderma amboinense*, have also demonstrated efficacy against liver disease. This species has shown potential action in liver disorders. According to earlier studies, *Ganoderma amboinense* polysaccharide (GAP) has protective effects on the liver [73]. In one research study, GAP was administered to high-fat-diet (HFD) mice for 8 weeks in order to assess GAP’s potential to prevent NAFLD and investigate its mode of action. The findings demonstrated that GAP effectively delayed the onset of NAFLD while also lowering blood lipid levels, liver weight, body weight and liver mass. By controlling the phosphatidylcholine content in the serum and metabolomics analysis, it was discovered that GAP increases fat transfer in the liver [74]. Simultaneously, GAP also controlled certain metabolic pathways as well as protects HFD mouse liver cells’ mitochondrial function, which led to rapid lipid catabolism. These outcomes showed that GAP could be applied as a potent preventive as well as therapeutic agent, alone or in combination with other therapeutic agents for the management of NAFLD [74]. Its multifaceted mechanisms are depicted in Figure 3.

### 5.4. Protective Effects against Hepatic Carcinoma

Cancer has become a growing global public health concern. In developed nations, it is the most common cause of death [75]. *G. lucidum* chemical compounds have anticancer activities primarily through multiple pathways, such as host immunomodulators, cytotoxic properties, the induction of metabolizing enzymes, etc. Among the numerous compositions of GLPS and triterpenoids, *G. lucidum* is being studied extensively due to numerous studies in which it has shown effects on cancer [6,8].

Hepatocellular carcinoma (HCC) is one of the world’s most dangerous cancers [76]. Chemoembolization and systemic therapies for HCC remain ineffective due to chronic liver infection and inflammation. Several studies have discovered an upsurge in CD4+ CD25+ regulatory T cells (Tregs) in both peripheral blood and the tumor microenvironment in HCC patients, which coincides with a poor prognosis [38,77]. Tregs impair the anti-tumor immune reaction and assist tumor cells in evading cellular immunity [78]. Managing the number and performance of Tregs may therefore be a valuable and successful HCC therapeutic strategy. The effect of GLPS on the balance of regulatory T cells (Treg) and effector T cells (Teff) in hepatoma-bearing mice has been studied. In hepatoma-bearing mice, GLPS significantly inhibits tumor growth, which is associated with an increase in the ratio of Teffs to Tregs. Furthermore, GLPS inhibits Treg’s suppression of Teff’s proliferation while increasing IL-2 secretion. A GLPS treatment of T lymphocytes reduced the expression of FoxP3 and Notch1 by increasing miR-125b expression [79].

The effects of *G. lucidum* fruiting body dry extract on peripheral blood lymphocytes and human liver tumor cells (HepG2/C3A) were assessed. It was discovered that fruiting body dry extract exhibited toxic effects to tumor cells, reducing their viability by causing DNA damage and boosting their production of ROS. In contrast, fruiting body dry extract was hazardous to lymphocytes only at high doses and reduced their viability, whereas at low quantities, it improved lymphocyte viability. Furthermore, *G. lucidum* fruiting body dry extract only caused primary DNA damage at the highest measured dose. As a result, *G. lucidum* exhibits cytotoxic and genotoxic activity as well as possible anticancer effects on malignant liver cells [8]. In order to alter the tumor microenvironment, *G. lucidum* spore polysaccharide (GLSP) activates macrophages, controls their polarization and encourages the discharge of numerous inflammatory mediators and cytokines [8]. Additionally, it has been discovered that GLSP activates macrophages to block H22 tumor cells in the G2/M phase as well as PI3K/AKT signaling pathways to influence the mitochondrial apoptotic pathway and increase tumor cell death. Apoptosis and autophagy are significant molecular developments that preserve organismal and cellular homeostasis, respectively. Although autophagy preserves cellular homeostasis by recycling specific intracellular organelles and chemicals, apoptosis performs its function by destroying diseased or undesirable cells [80]. Therefore, GLSP, a naturally occurring vitamin, has the ability to change macrophage polarity and potentially affect the activity of the tumor microenvironment [57,81].

**Figure 4:**
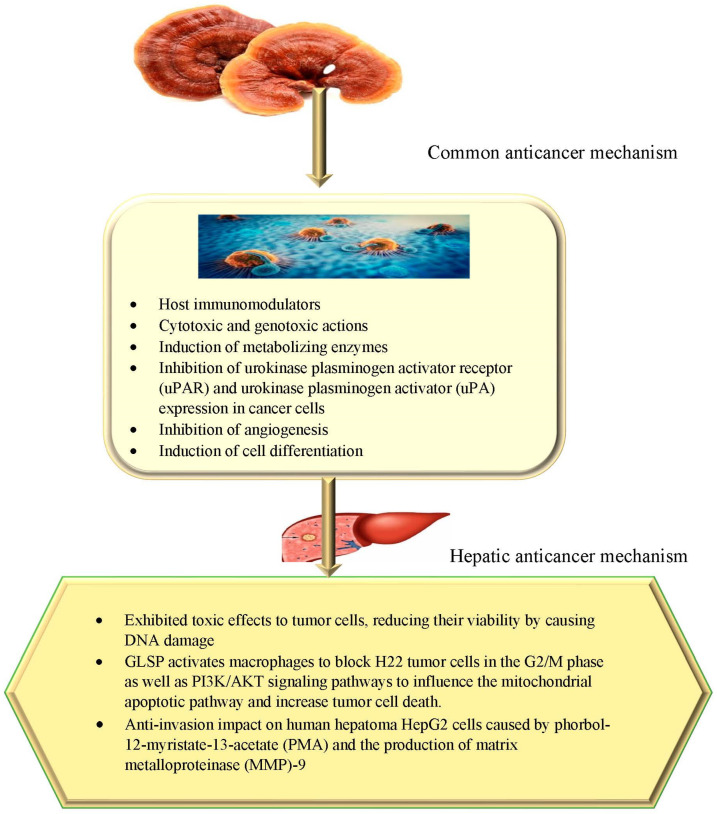
Proposed mechanism of *G. lucidum* against hepatic cancer.

Moreover, the anti-invasion impact of GLE on human hepatoma HepG2 cells was assessed [82]. HepG2 cell invasion caused by both phorbol-12-myristate-13-acetate (PMA) and the production of matrix metalloproteinase (MMP)-9 was prevented by GLE management in a dose-dependent manner. GLE inhibited ERK1/2 and the phosphorylation of protein kinase B in the cytoplasm, along with nuclear factor-κB and activator protein-1 levels in the nucleus of HepG2 cells, which all contributed to the inhibitory effects of GLE on MMP-9 production [56]. The suppression of the dosage response in terms of tumor size, volume and weight on average was observed in a human tumor xenograft model after the oral administration of GLE. The oral treatment of GLE considerably reduced the quantity of mice with metastatic tumors, the quantity of affected organs, the number of tumor foci, and the MMP-2 and -9 actions in mouse serum. These findings indicate that the highly invasive hepatoma cells’ tumorigenesis and metastasis could be prevented by lucidenic acid-rich GLE [56,82]. The mechanisms are depicted in Figure 4.

### 5.5. Protective Effect against Carbon Tetrachloride

The CCl4 metabolite trichloromethyl free radical is the major cause of hepatotoxicity. It binds with tissue macromolecules and then encourages membrane lipid degradation and finally damages the membrane. It is expected that such progress tends toward lipid peroxidation [83,84,85]. According to Sancheti et al. (2013), the liver’s metabolism of CCl4 produces free radicals, which in turn cause oxidative stress, a combined pathogenic mechanism that progresses liver damage [86,87]. Hepatocytes undergo apoptosis, necrosis, inflammation and the development of liver fibrogenesis and fibrosis [88]. In response, oxidative stress triggers the release of inflammatory cytokines [88,89]. It has been reported that the levels of MDA, SOD, H_2_O_2_ content, GSH and CAT were restored after treatment with GLE, preserving the enzymes [90]. *G. lucidum* is a potent antioxidant that significantly inhibits the elevated MDA level and demonstrates substantial free radical scavenging activity [91,92].

**Figure 5:**
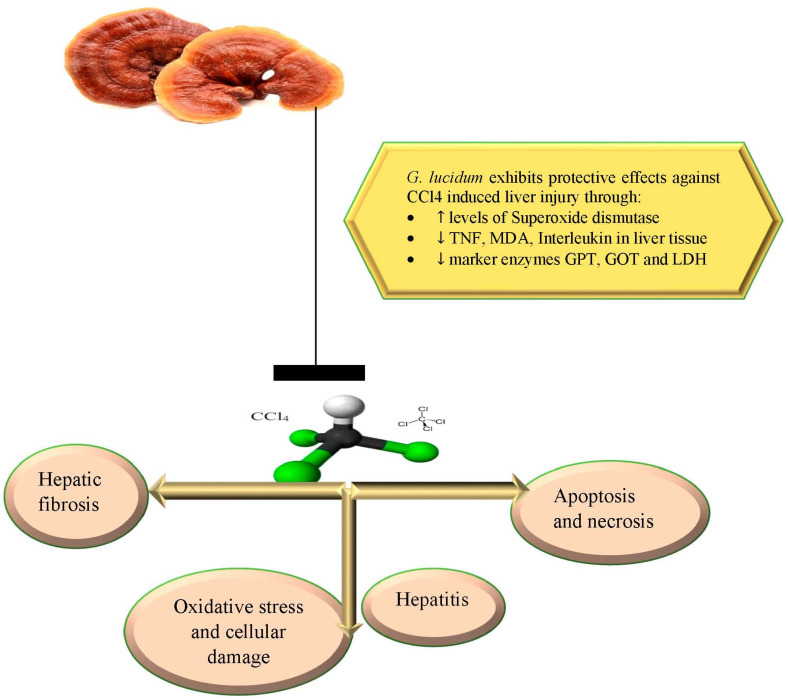
*G. lucidum* exhibits potential action against CCl4-induced liver injury through regulating different enzymes and scavenging free radicals.

In one study, GLPS was found to have hepatoprotective effects on common carp hepatocyte injury brought on by CCl4. According to the findings, GLPS greatly boosted cell viability, suppressed the elevations of the marker enzymes (GOT, GPT and LDH), and MDA caused by CCl4, and dramatically increased the levels of SOD. The expression of CYP1A and CYP3A was markedly downregulated during the GLPS treatments, along with extrinsic apoptosis and the immunological inflammatory response. GLPS can prevent hepatocyte injury brought on by CCl4 by decreasing lipid peroxidation, increasing the activities of antioxidant enzymes, and suppressing apoptosis and the immunological inflammatory response [18]. Additionally, GPLS was found to have anti-inflammatory and hepatoprotective properties against CCl4-induced liver injury in Kunming mice [52]. A further similar study also found that treatment with GA at 10 mg and 30 mg/kg for seven days significantly protected Kunming mice from liver damage produced by carbon CCl4 [61]. Anti-inflammatory and hepatoprotective effects of GLPS along with potential mechanisms have been reported in mice with acute liver injury caused by CCl4. In mice with liver injury, GLPS dramatically reduced the activation of the NLRP3 inflammasome and enhanced liver function. It remarkably reduced liver weight, interleukin (IL)-1, 18, and 6, total bilirubin, TNF, MDA, and IL-1 in serum, as well as MDA in liver tissue, which were all markedly repressed by CCl4-induced changes in ALT and AST activities in serum. While the GSH content in hepatocytes was noticeably increased by GLPS, the expression of protein levels in the liver, such as ASC, NLRP3 and caspase-1, was reduced [52]. *G. lucidum*’s effects are depicted in Figure 5.

### 5.6. Protective Effect against α-Amanitin-Induced Liver Injury

The majority of deadly mushroom poisonings are brought on by Amanita species; these mushrooms contain amatoxins that cause acute liver failure. A distinguished traditional healing mushroom, *G. lucidum*, exhibits hepatoprotective properties against such toxicities [58]. *G. lucidum* triterpenoids’ hepatoprotective effects on liver damage by α-amanitin (α-AMA) in rats were examined, and significant effects were reported through radical scavenging and antiapoptotic properties. The mice were treated with and monitored by total or individual triterpenoids of *G. lucidum*, and the triterpenoids’ hepatoprotective impacts were evaluated by comparing them with silibinin (SIL) [37,93]. The SIL treatment with *G. lucidum*’s total triterpenoids decreased death rates by 20–40% and considerably reduced the serum levels of ALT and AST. In addition, triterpenoids and SIL dramatically increased catalase and SOD activity and decreased MDA levels in the mice livers. The treatment with GA-C2 dramatically reduced caspase-3, -8, and -9 activity and significantly suppressed DNA fragmentation. The findings showed that triterpenoids have hepatoprotective effects on liver injury induced by α-AMA, and that these benefits may be caused by their antioxidative radical-scavenging properties as well as their prevention of apoptosis [37].

The effects of GLE on liver damage by α-AMA were also examined in a similar investigation, as well as potential mechanisms for hepatoprotection linked to radical scavenging activity. Mice were given an injection of α-AMA made from *Amanita exitialis*, followed by the administration of GLE. A reference medicine, SIL, was used to compare GLE’s hepatoprotective activity. The effects of α-AMA included a considerable increase in the serum levels of ALT and AST, as well as a substantial decline in the activities of the antioxidant enzymes’ catalase and SOD. When compared to the α-AMA control group, the treatments with GLE or SIL considerably reduced serum AST and ALT levels, considerably boosted the actions of CAT and SOD, and significantly diminished the MDA content in the liver [58].

### 5.7. Protective Effects against Hepatitis B Virus

Hepatitis is an inflammation of the liver, and the liver is an important organ for filtering blood [16]. It distributes nutrients and protects organisms from infections. Inflammatory responses or damage to the liver can impair its function. Hepatitis can be influenced by chemical compounds, drugs, certain medical conditions and excessive alcohol consumption. Hepatitis B is an infection caused by the hepatitis B virus (HBV), which can result in both acute and chronic infections. Cirrhosis and liver cancer may eventually appear, having a detrimental impact on people’s health, even though the majority of those with HBV infections show no symptoms [94,95]. It has been established that GAs belong to class of bioactive compounds found in *G. lucidum* [96], which exhibit a potential role in inhibiting the replication of HBV. The replication of the hepatitis B virus (HBV) in HepG2215 cells was suppressed for eight days by GA from *G. lucidum* at a concentration of 8 µg/mL. The mice were also significantly protected by GAs from liver damage caused by *M. bovis* BCG and lipopolysaccharide (from *Escherichia coli* 0127:B8) [61,95]. Moreover, the liquid fermentation broth of *G. lucidum* was tested for anti-HBV and hepatoprotective action. Radix *Sophorae flavescentis* aqueous extract, a Chinese herbal medicine, was added to the cultured broth. In vitro, the cultured broth exhibited anti-HBV activity and protected mice from hepatic injury. Additionally, it has been claimed that co-fermenting *G. lucidum* broth with Radix *Sophorae flavescentis* aqueous extract results in stronger therapeutic effects than merely combining these two components [61].

## 6. Effects on Microbiota and Latest Findings

Excessive alcohol intake is one of the foremost causes of intestinal microbial ailments, which have been meticulously linked to the pathogenesis of hepatic diseases [97]. It has been hypothesized that consuming large amounts of alcohol has a significant impact on the flora in the intestinal tract [98]. In mice consuming large amounts of alcohol, GLE interventions drastically altered the gut microbial ecology. An earlier study demonstrated that GLE intervention changed the intestinal microbiota composition in mice subjected to high-fat diets [99]. The oral delivery of GLE could alter the intestinal microbiota composition in mice consuming excessive amounts of alcohol, according to a hierarchical clustering study. By boosting the numbers of *Faecalibaculum*, *Lactobacillus*, *Bifidobacterium* and *Romboutsia* and lowering the level of *Helicobacter*, GA intervention altered the composition of intestinal microflora, as shown by intestinal microbiota profiling. In addition, liver metabolomic profiling indicated that GA intervention had a notable regulatory influence on liver metabolism after drinking too much alcohol [68]. Moreover, water-soluble polysaccharide obtained from *G. lucidum* spores exhibited substantial effects against cancer and altered gut microbiota caused by AOM/DSS. The spores increased goblet cells, MUC2 production and tight junction protein expression. They also significantly enhanced gut barrier function. In addition to reducing the expression of IL-1, iNOS, and COX-2, GLP therapy also prevented macrophage infiltration. Additionally, GLP reduced the inflammatory markers in macrophage RAW264. 7, intestinal NCM460, HT-29 cells and the activation of mitogen-activated protein kinases. These findings suggest that GLP is a viable prebiotic for the management of colorectal cancer [100].

The crude polysaccharide of *G. lucidum* was discovered to have a defensive effect on liver injury due to H_2_O_2_ stress in mice by enhancing oxidative status. Two different polysaccharides purified from GLP, acidic-glucan (GLPC2) and neutral-glucan (GLPB2), exhibited a stronger hepatoprotective effect against H_2_O_2_-induced liver injury in HepG2 cells [101]. As a proven universal mechanism for cell and tissue destruction, cellular oxidative damage is predominantly caused by ROS. Through enhancing the oxidative state, GLP demonstrated a protective role against acute hepatic injury produced by constraint stress. Hydrogen peroxide is a hazardous material that can be transformed into hydroxyl and oxygen radicals by hepatocytes. Prior studies had revealed that polysaccharides had hepatoprotective impacts depending on the viability, ALT and AST behavior of H_2_O_2_-induced HepG2 cells [102]. Hydrogen peroxide induced an upsurge in ALT and AST actions in HepG2 cells. GLPB2 and GLPC2 significantly prevented ALT and AST activities in a concentration-dependent manner. Furthermore, GLPC2 had more potent inhibitory effects than GLPB2. The presence of glucuronic acid in GLPC2 may contribute to its better hepatoprotective effects. According to ongoing studies, polysaccharides can protect the liver through a number of different mechanisms of action, such as regulating apoptosis and oxidative stress. Apparently, JAK/STAT, NF-kB, TGF-β, MAPK, PI3K/AKT, caspase cascade, Nrf2-Keap1 pathways, cytochrome P450 enzymes, and lipid metabolism can be regulated by polysaccharides [101,102]. In order to confirm the hepatoprotective activity of GLPC2 in vivo and to understand the underlying mechanism, more research is required. The findings established a theoretical foundation for the potential application of GPLS as a hepatoprotective substance in the food and pharma industries [101]. Acute liver failure is most frequently caused by drug-induced liver damage. *G. lucidum* protects from drug-induced hepatic injury through inhibiting HMGB-1/NF-kB and caspase-3. It modulates oxidative stress and the ensuing cross-talk between the inflammatory and apoptotic cascades, revealing its potential contribution to drug-induced hepatic injury. In addition, the *G. lucidum* mushroom also exerts hepatoprotective effects against cadmium and tert-butyl hydroperoxide (t-BHP)-induced hepatic injury [62].

## 7. Concluding Remarks

*G. lucidum* is a traditional medicinal and edible mushroom that has a significant role in preserving human health. The demand for this mushroom is rising across the world as a medicinal, nutraceutical and functional food. The efficient hepatoprotective action of the naturally occurring, biologically active compounds confined to *G. lucidum* is promising in the endeavor to find successful hepatoprotective substances. Novel drugs from natural sources that can be alternatives to synthetic medicine with fewer side effects are needed. As a result, these investigations offer insightful information and a solid foundation for developing new medications to treat hepatic disorders from *G. lucidum*. Because of the global trend of an increase in the number of people with liver ailments, there is substantial demand for effective medications that can provide potential outcomes through multifaceted mechanisms. Moreover, research and clinical trials are in progress to determine the efficacy of numerous compounds obtained from *G. lucidum* in support of hepatoprotective activity. Studies and more research could make it easier to create medicinal and nutraceutical formulations that could be used to treat a wide range of diseases, particularly hepatic cancer, hepatitis, hepatic fibrosis and NAFLD. Additional investigations are needed through different parameters to find out the various unrevealed compounds that could be used in further experimental and clinical studies to eradicate hepatic disorders. 

## Figures and Tables

**Table 1:** Hepatoprotective actions of *G. lucidum* in different liver disorders.

Hepatic Injury	Different Form of Constituents	Hepatoprotective Effects	References
Hepatic injury induced by alcohol	*G. lucidum* ethanol extract	Inhibits high levels of serum TG, TC, low-density lipoprotein cholesterol (LDL-C), AST and ALT Decreases the level of MDA and lactate dehydrogenase (LDH) Increases the levels CAT, GSH, and SOD as well as alcohol dehydrogenase	[51]
Carbon tetrachloride (CCl4)-induced liver injury	*G. lucidum* polysaccharides	Boosts cell survival and prevents an increase in the marker enzymes GPT, GOT and LDH caused by CCl4 Effectively increases the levels of superoxide dismutase It significantly reduces liver weight, total bilirubin, interleukin (IL)-1, IL-18, IL-6, TNF, MDA, IL-1 in serum, IL-1 and MDA in liver tissue	[18,52]
	Crude polysaccharides extract	The contents of MDA, ALT and AST are greatly reduced by the *G. lucidum* extract, whereas the amounts of SOD and CAT are markedly elevated The histopathology of liver tissue, such as hydropic degeneration and necrosis, is also improved	[40]
	*G. lucidum* sporoderm-breaking spores	Significantly decreases the deposition of hepatic collagen in CCl4-induced hepatic fibrosis rats Reduces TGF-1 and TIMP-1 expression, prevents the synthesis of collagen, and boosts the decomposition of collagen	[50]
	*G. lucidum* Spores oil	In CCl4-induced liver injury model mice, AST and ALT activity dramatically decrease	[39]
Concanavalin A (CON A)-induced immune liver injury	Broken *G. lucidum spores* powder	When compared to the model group, the mice exposed to broken *G. lucidum* spore powder have significantly lower serum levels of ALT and AST, as well as a significantly lower the rate of liver inflammatory cell degeneration and necrosis	[41]
Galactosamine-induced liver fibrosis effects	*G. lucidum* triterpene extract	￭ The hepatoprotective concern of *G. lucidum* triterpene extract may be linked to enzyme actions that lead to counteracting free radicals ￭ Lowers MDA and hydrogen peroxide levels, boosts GSH levels and antioxidant enzyme action, and maintains the normal range of nitrite ￭ Improves antioxidant ability	[53,54]
Hepatic fibrosis	*Ganoderma applanatum* (triterpenoids) Ganoapplanic acid A, C, F	￭ Inhibits the proliferation of hepatic stellate cells (HSCs)	[55]
	*G. lucidum* spores powder	￭ It considerably reduces the fibrosis of mouse liver brought on by CCl4. The serum MDA, ALT, and AST levels as well as the levels in hepatic tissue are much lower in the *G. lucidum* spores powder group than in the other group, and the expression of the MMp-9 protein in hepatic tissue is dramatically reduced	[50]
Hepatic carcinoma	*G. lucidum* extract (GLE)	￭ It is revealed that GLE exhibits toxic effects on tumor cells, reducing viability through DNA impairment ￭ Inhibitory effects produced by GLE on MMP-9 production	[8,56]
	*G. lucidum* spore polysaccharide	GLSP triggers macrophages and boosts the discharge of a range of inflammatory mediators and cytokines GLSP triggers macrophages to block H22 tumor cells in the G2/M phase as well as PI3K/AKT signaling pathways to influence the mitochondrial apoptotic pathway and increase tumor cell death	[57]
α-Amanitin Induced Liver Injury	*G. lucidum* aqueous extracts	Lowers the elevated levels of ALT and AST Significantly reduces MDA content in liver	[37,58]
	Ganoderic acid C2	Considerably reduces the DNA fragmentation and decreases caspase 3,-8 and 9 actions	[37]
Non-alcoholic fatty liver disease	Fudan-Yueyang *G. lucidum* (FYGL)	Decreases TC and TG levels in hepatocytes Increases the action of enzymes acetyl-CoA carboxylase (ACC) and AMP-activated protein kinase (AMPK) Prevents steatosis induced by the oxidation of fatty acids by increasing the expression of carnitine palmitoyl transferase-1 (CPT-1) FYGL decreases ROS and MDA and boosts total antioxidant capacity and SOD GLPP considerably recovers NAFLD through the regulation of the synthesis of bile acid dependent on FXR-SHP/FGF pathway	[59,60]
Hepatitis B	Ganoderic acids	Inhibits the replication of hepatitis B virus	[61]
Drug induced liver injury (Cisplatin)	*G. lucidum* mushroom (GLM)	Decreases ALT, AST and total bilirubin, as well as oxidative stress markers MDA and H_2_O_2_ Inhibits HMGB-1/NF-kB and caspase-3 Increases hepatic GSH and SOD	[62]
Formaldehyde (FA) induced liver fibrosis	*G. lucidum* ethanol extract	Decreases TNF, IL-1 and IL-6 Lowers MDA and hydrogen peroxide levels Boosts the GSH and antioxidant enzymes Maintains normal range of myeloperoxidase formation	[17,63]
Obstructive jaundice	*G. lucidum* polysaccharide	Reduces the aberrant levels of bilirubin, protein carbonyl, MDA, thiol and GSH in the plasma and liver of the common bile duct-ligated rat	[64]

## Data Availability

Not applicable.
